# Prediction of hematologic toxicity in luminal type breast cancer patients receiving neoadjuvant chemotherapy using CT L1 level skeletal muscle index

**DOI:** 10.1038/s41598-024-58433-9

**Published:** 2024-04-13

**Authors:** Min Chen, Pinxiu Wang, Yanting Li, Zhuanmei Jin, Yu An, Yanan Zhang, Wenzhen Yuan

**Affiliations:** 1 The First Clinical Medical College, Lanzhou University, Lanzhou, 730000 Gansu Province China; 2 Department of Oncology, Shucheng People’s Hospital, Lu’an, 231300 China; 3 The Department of Oncology, The First Hospital of Lanzhou University, Lanzhou, 730000 Gansu Province China

**Keywords:** Breast cancer, Gynaecological cancer, Cancer, Diseases, Health care, Medical research, Oncology, Risk factors

## Abstract

This study aims to explore the correlation between the CT-L1 and L3 body composition parameters and analyze the relationship between L1 body composition and hematologic toxicity in luminal-type breast cancer patients undergoing neoadjuvant chemotherapy. Data from 140 luminal-type breast cancer patients who underwent surgical treatment after neoadjuvant chemotherapy were analyzed retrospectively. Spearman analysis was used to assess the correlation between CT-L1 and CT-L3 body composition parameters pre-neoadjuvant chemotherapy. Additionally, univariate and multivariate logistic regression analyses were performed to identify factors influencing hematologic toxicity. CT-L1 body composition parameters were positively correlated with CT-L3 body composition parameters in 34 patients. Severe hematological toxicity occurred in 46 cases among the patient cohort. A skeletal muscle index (SMI) of < 32.91 cm^2^/m^2^, initial tumor size ≥ 3.335 cm, and a glucose-to-neutrophil ratio (GLR) ≥ 2.88 were identified as independent risk factors for severe hematologic toxicity during neoadjuvant chemotherapy in luminal-type breast cancer patients. The sample size in this study is small, and the predictive capacity of GLR in hematologic toxicity requires further research for comprehensive validation. CT-L1 analysis represents a viable alternative to CT-L3 analysis for body composition assessment. Patients with a low skeletal muscle index were more prone to experiencing severe hematologic toxicity during neoadjuvant chemotherapy.

## Introduction

Breast cancer (BC) is a globally prevalent malignancy, accounting for approximately 30% of malignant tumors in females^1^. Some BC patients require neoadjuvant chemotherapy (NACT) before surgery, which plays a crucial role in reducing the tumor stage^2^. Hematological toxicity represents a common adverse effect of NACT. Severe hematological toxicity can lead to compromised immune function, heightened risks of infection and bleeding, and potentially life-threatening consequences. In addition, failure to adequately address pronounced hematological toxicity may impede the progress of antitumor therapy, posing obstacles to the smooth implementation of anticancer treatment. Presently, the NACT drug dosage is determined based on the body surface area (BSA). However, body composition, including muscle and fat distribution areas, is significantly correlated with patient tolerance and chemotherapy toxicity levels^3^. For instance, a study on non-metastatic BC patients treated with anthracyclines and taxanes revealed a lower probability of hematologic toxicity in patients with higher muscle mass^4^. Thus, body composition may serve as a predictive indicator of hematologic toxicity, enabling personalized adjustment of chemotherapy drug dosages based on differences in body compositions of patient^5,6^. This personalized approach could mitigate adverse reactions to chemotherapy.

Current investigations on body composition predominantly rely on data obtained from abdominal CT (computed tomography, CT) scans at the third lumbar vertebra (L3) level. However, routine chest CT examinations for most BC patients typically are conducted only at the first lumbar vertebra (L1) level. Consequently, the inability to access L3 level measurements for the majority of BC patients impedes effective body composition assessment. Could chest CT scans be conducted at an alternative level that could substitute for L3? Researchers have initiated preliminary inquiries in this regard. For instance, in chronic obstructive pulmonary disease patients, research suggests that the pectoralis major muscle could act as a surrogate for assessing muscle content at the L3 level^7^. Additionally, in individuals undergoing routine health check-ups, a separate study revealed the feasibility of using CT-L1 level measurements as a substitute for quantifying total body muscle and fat content typically assessed at the L3 level^8^. Moreover, Karin et al.^9^ showed that CT-L1 level assessments could replace body composition evaluations typically conducted at the CT-L3 level in non-small cell carcinoma patients.

Presently, there is no consensus on establishing an alternative level for evaluating body composition in BC patients, with the exception of CT-L3. Thus, our primary objective was to investigate the correlation between body composition measurements at the CT-L1 and CT-L3 levels in these patients. Furthermore, our study aimed to determine whether body composition metrics, specifically fat content and skeletal muscle content, assessed pre-NACT at the L1 level, are associated with hematologic toxicity during NACT. Given the diverse clinical manifestations, treatment modalities, adverse reactions, and prognostic implications observed across distinct molecular subtypes of BC^10^, our research specifically focuses on patients with luminal-type BC as the focal cohort for examination.

## Materials and methods

### Study population

The study included patients diagnosed with primary BC who underwent NACT followed by surgical intervention at the First Hospital of Lanzhou University between July 2018 and September 2023. Inclusion criteria were: (1) luminal-type breast cancer pathology; (2) unilateral breast cancer in females; (3) completion of 4–8 cycles of NACT; (4) underwent chest and/or abdominal CT examinations before NACT. Exclusion criteria were: (1) anti-tumor therapy administration before diagnosis; (2) pregnancy or lactation; (3) coexistence of other malignancies from different tissue origins; (4) missing essential data.

### Clinical data collection

Patient information encompassed demographics (age, height, weight, menopausal status, body mass index (BMI = weight divided by the square of height), pathological details (pathological type, axillary lymph node metastasis status, pre-and post-NACT tumor size, immunohistochemistry results), NACT regimen, and hematologic parameters during NACT (white blood cells, neutrophils, hemoglobin, platelets, lymphocytes). To consider the potential impact of biochemical markers such as glucose and lymphocytes on hematologic toxicity, we determined the neutrophil-to-lymphocyte ratio and the glucose-to-lymphocyte Ratio (GLR) in our investigation. Body composition metrics comprised skeletal muscle area (SMA), SMI = skeletal muscle area divided by the square of the height), intermuscular fat area (IMFA), intermuscular fat index (IMFI = intermuscular fat area divided by the square of the height), subcutaneous fat area (SFA), subcutaneous fat index (SFI = subcutaneous fat area divided by the square of the height), visceral fat area (VFA), and visceral fat index (VFI = visceral fat area divided by the square of the height). Optimal cutoff values derived from ROC curves were applied to dichotomize continuous variables. CT images were analyzed at the CT-L1 and L3 levels using Slice-O-matic software to quantify body composition metrics (muscle, visceral fat, subcutaneous fat, intermuscular fat). Sarcopenia was defined in accordance with the clinical practice guidelines of the European Liver Research Association as a female skeletal muscle index (SMI) < 39 cm^2^/m^2^ at the L3 level^11^. Nonetheless, no unified definition exists in East Asia. Prof. Zhen Yu proposed sarcopenia as a female SMI < 34.9 cm^2^/m^2^ at the L3 level in gastric cancer patient^12^. Given the racial, dietary, genetic, and economic variations among European, American, and Chinese populations, as well as the partial differences in body composition between L1 and L3 levels, patients with an SMI lower than the determined cutoff value were categorized into the sarcopenia group after preliminary experimentation.

### Hematologic toxicity assessment

Hematologic toxicity, including anemia, leukopenia, neutropenia, and thrombocytopenia, was graded according to the radiation therapy oncology group (RTOG)^13^ criteria. Severe hematologic toxicity was defined as grade 3 or higher (e.g., hemoglobin < 80 g/L, white blood cells < 2.0 × 10^9^/L, neutrophils < 1.0 × 10^9^/L, platelets < 50 × 10^9^/L). In cases of multiple grades of anemia, leukopenia, neutropenia, or thrombocytopenia, the highest grade was considered.

### Statistical analysis

Statistical analyses were performed using SPSS software with significance set at P < 0.05. The receiver operating characteristic curve (ROC) curve analysis was used to determine optimal cutoff values for continuous variables. Spearman correlation analysis helped to evaluate relationships between body composition parameters at the CT-L1 and L3 levels, with correlations of r > 0.5 considered positive and r > 0.7 indicating strong correlations. Variables with P < 0.05 in the univariate analysis were included in the multivariate logistic regression analysis.

### Ethical information

The registration table data was approved by the first hospital ethics committee of Lanzhou University (LDYY-2023–524), and all experiments were performed in accordance with relevant guidelines and regulations. The first hospital ethics committee of Lanzhou University has exempted informed consent.

### Ethical review

The registration table data was approved by the first hospital ethics committee of Lanzhou University (LDYY-2023-524).

### Waive the informed consent statement

This is a retrospective study: 1. The medical records or biospecimens used in this study were obtained during previous clinical consultations; 2. The waiver of informed consent will not adversely affect the rights or health of the subjects; 3. The privacy and personally identifiable information of the subjects will be protected. The first hospital ethics committee of Lanzhou university has exempted informed consent.

## Results

### Baseline characteristics

The study included a cohort of 140 luminal-type BC patients (Table 1), with an average age of 49.4 years (ranging from 25 to 74 years), with 68 patients aged ≤ 49 years and 72 patients aged > 49 years. Among these patients, 82 were postmenopausal, while 58 were premenopausal. A total of 60 patients had a body mass index (BMI) ≥ 24 kg/m^2^, while 80 patients had a BMI < 24 kg/m^2^. Among all individuals, there were 6 patients underweight, 74 normal weight, and 61 overweight. Notably, 98 patients exhibited initial tumors < 3.335 cm, while 42 patients presented tumors ≥ 3.335 cm. Axillary lymph node metastasis was observed in 100 cases, and 40 cases did not show metastasis. A total of 59 patients exhibited an SMI < 32.91 cm^2^/m^2^, all of whom were diagnosed with sarcopenia. Hematologic parameters (hemoglobin, white blood cells, neutrophils, platelets) were considered as outcome variables. Among the total sample of 140 patients, there were 46 cases that ultimately experienced severe hematologic toxicity. Specifically, there were 40, 41, 1, and 4 cases of severe neutropenia, severe leukopenia, severe anemia, and severe thrombocytopenia observed within this cohort. Among these 46 patients, a total of 24 completed 8 cycles of NACT, 4 completed 7 cycles, 6 completed 6 cycles, 4 completed 5 cycles, and 8 completed 4 cycles. Among the 8 patients who received 7 or 5 cycles of NACT, 4 discontinued treatment prematurely due to severe hematologic toxicity, 2 due to disease progression, and the reasons for the remaining 2 cases were lost.

**Table 1:** Baseline characteristics of patients.

Pathological features	Groups	Number of cases	Percentage (%)
Age	≤ 49	68	48.57
	> 49	72	51.43
BMI (kg/m^2^)	< 24	80	57.2
	≥ 24	60	47.8
Menopausal status	Menopausal	82	58.57
	Non-menopausal	58	41.43
Initial tumor size (cm)	< 3.335	98	70.00
	≥ 3.335	42	30.00
Occurrence of axillary lymph node metastasis	Yes	100	71.43
	No	40	28.57
ER	−	3	2.14
	+	137	97.86
PR	−	32	22.86
	+	108	77.14
Ki67	−	23	16.43
	+	117	83.57
NLR	< 2.88	45	32.14
	≥ 2.88	95	67.86
GLR	< 1.86	42	30.00
	≥ 1.86	98	70.00
SMA (cm^2^)	< 87.32	70	50.00
	≥ 87.32	70	50.00
SMI (cm^2^/m^2^)	< 32.91	59	42.14
	≥ 32.91	81	57.86
IMFA (cm^2^)	< 3.59	27	19.29
	≥ 3.59	113	80.71
IMFI (cm^2^/m^2^)	< 1.35	27	19.29
	≥ 1.35	113	80.71
SFA (cm^2^)	< 155.25	103	73.57
	≥ 155.25	37	26.43
SFI (cm^2^/m^2^)	< 56.90	99	70.71
	≥ 56.90	41	29.29
VFA (cm^2^)	< 112.55	108	77.14
	≥ 112.55	32	22.86
VFI (cm^2^/m^2^)	< 34.78	88	62.86
	≥ 34.78	52	37.14
Whether dexamethasone used	Yes	54	38.5
	No	86	61.5
Whether severe hematological toxicity occurred	Yes	46	67.14
	No	94	32.86

### Relationship between body composition parameters at CT-L1 and CT-L3 levels

**Figure 1:**
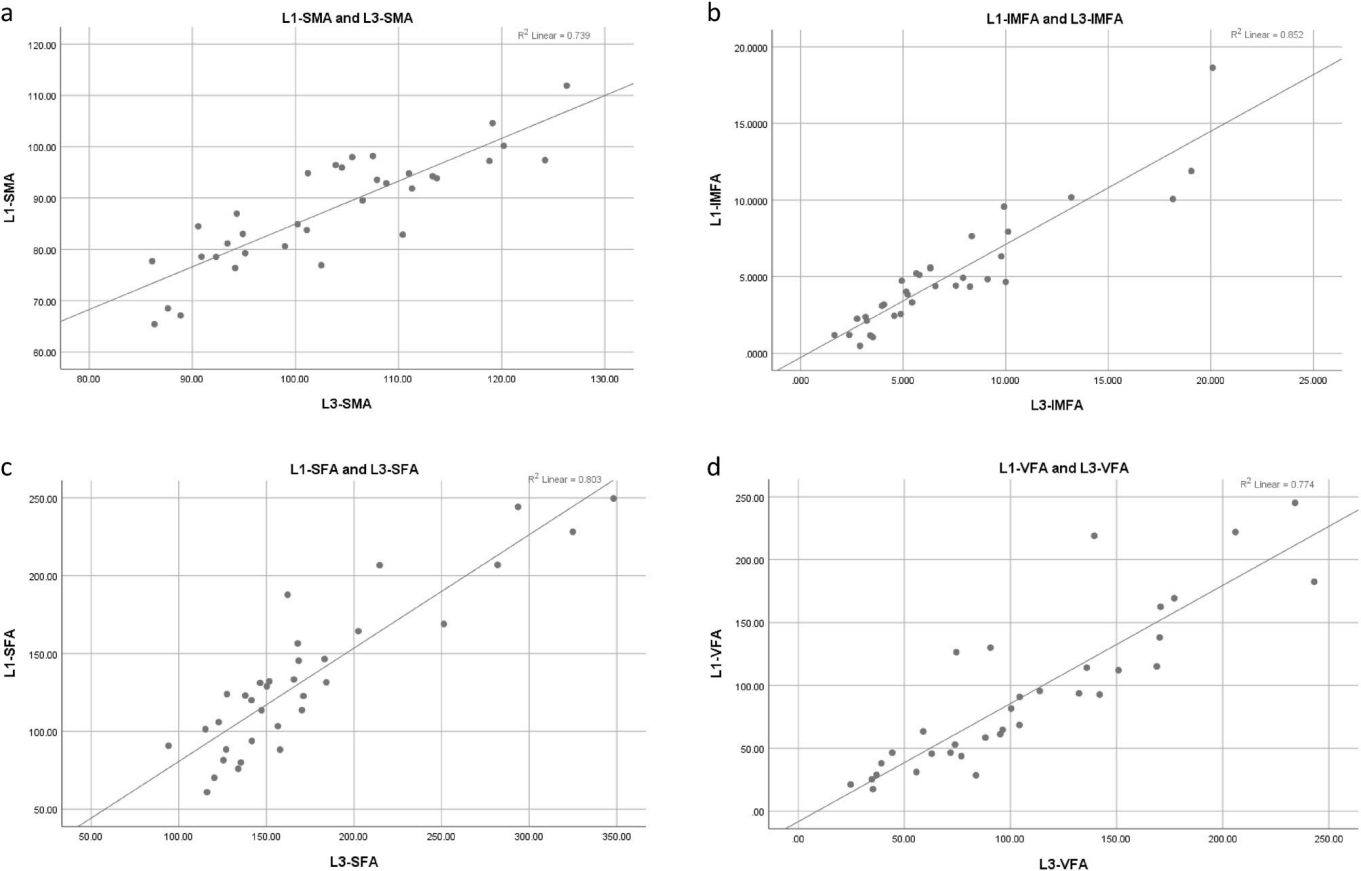
Scatter plots showing the correlation in body compositions between CT-L1 and CT-L3 levels. (**a**) Correlation between Skeletal Muscle Area (SMA) at the L1 and L3 levels; (**b**) Correlation between Intermuscular Fat Areas (IMFAs) at the L1 and L3 levels; (**c**) Correlation between Subcutaneous Fat Areas (SFAs) at the L1 and L3 levels; (**d**) Correlation between Visceral Fat Areas (VFAs) at the L1 and L3 levels.

Among the 140 luminal-type BC patients, a subgroup of 34 individuals underwent both chest and abdominal CT scans before starting NACT. Due to the potential collinearity among metrics derived from SMA, IMFA, SFA, and VFA normalized by the square of the height (SMI, IMFI, SFI, VFI), our comparative analysis focused solely on SMA, IMFA, SFA, and VFA between the L1 and L3 levels. Scatter plots were constructed using SPSS software to visualize the relationship between body composition metrics at the L1 and L3 levels, as shown in Fig. 1. Notably, a linear positive correlation was evident between the L1 and L3 levels for SMA, IMFA, SFA, and VFA. Spearman correlation analysis revealed correlation coefficients (r) of 0.825, 0.914, 0.844, and 0.895 for SMA, IMFA, SFA, and VFA, respectively, between the CT-L1 and CT-L3 levels, all demonstrating statistical significance with P-values < 0.001. These findings underscore a robust positive correlation in body composition metrics between CT-L1 and CT-L3 levels, with correlation coefficients (r) consistently exceeding 0.7 (Fig. 1).

### Relationship between body composition and hematologic toxicity at the CT-L1 level in luminal-type BC patients during NACT

Based on laboratory test results during chemotherapy, 46 patients exhibited severe hematologic toxicity, while 94 did not exhibit severe hematologic toxicity. Univariate analysis was conducted using the chi-square test/Fisher's exact probability test. The results of univariate analysis demonstrated the associations between patient BMIs, initial tumor sizes, GLRs, SMAs, SMIs, SFAs, SFIs, VFAs, and VFIs with hematologic toxicity during NACT, all with P-values < 0.05. Variables with P < 0.05 in the univariate analysis were included in a multivariate binary logistic regression analysis. There was a substantial correlation between SMAs and SMIs; SFAs and SFIs; and VFAs and VFIs, with correlation coefficients of 0.903, 0.994, and 0.984, all exceeding 0.7, indicating severe multicollinearity. SMI, SFI, and VFI alone were included in the multivariate binary logistic regression analysis. The study findings (Table 2) revealed that an initial tumor size ≥ 3.335 cm, GLR ≥ 2.88, and SMI < 32.91 cm^2^/m^2^ were independent risk factors for severe hematologic toxicity during NACT in luminal-type BC patients (Table 2).

**Table 2:** Relationships between body composition, related indices, and hematologic toxicity.

Pathological features	Groups	Univariate	Multivariate logistic regression analysis	
		p value	OR (95% CI)	p value
Age (years)	≤ 49	0.554		
	> 49			
BMI (kg/m^2^)	< 24	0.179		
	≥ 24			
Menopausal status	Menopausal	0.702		
	Non-menopausal			
Initial tumor size (cm)	< 3.335	0.041	2.279 (1.026–5.345)	0.048*
	≥ 3.335			
Occurrence of axillary lymph node metastasis	Yes	0.955		
	No			
ER	−	0.986		
	+			
PR	−	0.827		
	+			
Ki67	−	0.096		
	+			
NLR	< 1.86	0.138		
	≥ 1.86			
GLR	< 2.88	0.003	3.89 (3.474–10.265)	0.006*
	≥ 2.88			
SMA (cm^2^)	< 87.32	0.001		
	≥ 87.32			
SMI (cm^2^/m^2^)	< 32.9	< 0.001	0.32 (0.134–0.761)	0.01*
	≥ 32.91			
IMFA (cm^2^)	< 3.59	0.06		
	≥ 3.59			
IMFI (cm^2^/m^2^)	< 1.35	0.06		
	≥ 1.35			
SFA (cm^2^)	< 155.25	0.036		
	≥ 155.25			
SFI (cm^2^/m^2^)	< 56.90	0.01	0.766 (0.256–2.291)	0.633
	≥ 56.90			
VFA (cm^2^)	< 112.55	0.005		
	≥ 112.55			
VFI (cm^2^/m^2^)	< 34.78	0.008	0.49(0.187–1.289)	0.148
	≥ 34.78			

* denotes statistical significance (p < 0.05).

Considering the impact of dexamethasone on blood glucose fluctuations, we conducted a single and multiple binary logistic regression analysis again on the 86 patients who did not use dexamethasone. The final results are presented in the Supplementary Table 1, indicating that SMI and GLR are risk factors affecting severe hematologic toxicity during neoadjuvant chemotherapy in luminal-type breast cancer patients. The analysis results after excluding the use of dexamethasone showed no significant difference compared to those before exclusion, except for fluctuations in initial tumor size results. This may be associated with the reduction in sample size. We look forward to further research to validate the predictive ability of initial tumor size.

## Discussion

This investigation uncovered a linear positive correlation between body composition metrics (SMA, IMFA, SFA, VFA) at the CT-L1 and CT-L3 levels in the cohort. In comparison to luminal-type BC patients exhibiting a CT-L1 SMI ≥ 32.91 cm^2^/m^2^, those with a with a lower SMI were notably predisposed to experiencing severe hematologic toxicity during NACT. Additionally, patients presenting with larger initial tumors (≥ 3.335 cm) and higher GLRs (≥ 2.88) were significantly more susceptible to severe hematologic toxicity during NACT. These findings underscore the critical necessity for timely monitoring of blood routine parameters and implementation of corresponding measures during NACT to mitigate the incidence of severe hematologic toxicity.

CT technology is the primary modality for quantifying body composition. Research suggests that body composition data can be derived from three distinct CT scan regions: chest (encompassing T1 to L1), abdomen (spanning T10 to L4), and pelvis (covering L4 to L5)^14^, with abdominal L3 being the most widely utilized^15^. However, only the chest CT data is available for most BC patients and there is a lack of supplementary abdominal CT information. Hence, we analyzed the relationship between body composition at the CT-L1 and L3 levels, and our results revealed a linear positive correlation in luminal-type BC patients. This can be attributed to the anatomical similarity in muscle composition between the L1 and L3 levels, encompassing muscles such as the psoas major, erector spinal, quadratus lumborum, transversus abdominis, internal oblique, external oblique, and rectus abdominis. This observation confirms previously established knowledge. In line with our findings, Derstine et al.^16^ assessed sarcopenia in a healthy US populace using CT-T10 to CT-L5 levels, demonstrating L1 as a preferable alternative to L3. Similarly, Pickhardt et al.^15^ indicated that the body composition at the L1 level provides more accurate survival outcome predictions than L3 in studies involving a healthy US population.

Our subsequent findings highlighted the significant predictive capacity of the SMI at the CT-L1 level for assessing hematologic toxicity in luminal-type BC patients.

Sarcopenia is a clinical syndrome characterized by a progressive reduction in total body muscle mass, reduced muscle strength, and diminished muscle function^17^. Previous reports have validated that patients with malignant tumors and sarcopenia are predisposed to severe hematologic toxicity. For instance, metastatic colorectal cancer patients with sarcopenia exhibited heightened rates of grade 3 and 4 hematologic toxicity post-chemotherapy^18^. Studies on postoperative adjuvant chemotherapy in BC patients showed that individuals with sarcopenia often exhibited more severe hematologic toxicity^19^. However, no studies exploring the link between sarcopenia and hematologic toxicity during NACT in BC patients have been conducted. The underlying cause contributing to the heightened predisposition of sarcopenic patients to severe hematologic toxicity during NACT remains elusive. It could be potentially attributed to chemotherapy dosages solely being determined by the BSA of the patient without considering the total body fat to muscle ratio. For instance, despite some patients exhibiting a BMI indicative of obesity, their skeletal muscle mass might have dwindled to a sarcopenic state. Consequently, if chemotherapy dosages are computed based on BSA for such patients, the dosage might surpass their tolerance threshold, resulting in severe hematologic toxicity^20,21^.

Furthermore, the size of the primary tumor emerged as an independent risk factor for severe hematologic toxicity during NACT in luminal-type BC patients. Larger initial tumors (≥ 3.335 cm) signify a more substantial tumor burden. Tumor metabolism and growth tend to exhaust body nutrients, especially by accelerating protein breakdown, thereby reducing muscle and fat content while compromising immunity, rendering patients more susceptible to severe hematologic toxicity during NACT. This is in alignment with the findings of John et al.^22^. Furthermore, as tumor volume escalates, inadequate tumor oxygenation and anaerobic glycolysis result in the accumulation of metabolic waste products such as lactate, consequently fostering a tumor microenvironment that suppresses the immune system^23^.

Additionally, while the GLR has prognostic significance for the detection of various malignant tumors^24,25^, its role in predicting chemotherapy-related hematologic toxicity has not been reported. Our findings showed that a high GLR value may also be a risk factor influencing severe hematologic toxicity. This mechanism might result in higher blood glucose and/or lower lymphocyte counts, which compromise the body's tolerance to chemotherapy. A previous study shows a 32% higher likelihood of neutropenia development in cancer patients with elevated blood glucose compared to those with normal blood glucose levels^26^. Furthermore, individuals with elevated blood glucose or diabetes are more predisposed to sarcopenia^27^, which reduces tolerance to chemotherapy. Moreover, diminished lymphocytes signify an immune deficiency. In studies on chemotherapy for colorectal cancer patients, reduced lymphocytes predicted poorer chemotherapy efficacy and increased hematologic toxicity^28^. Additionally, elevated blood glucose can reduce peripheral blood lymphocyte counts^29^, while effective blood glucose control enhances immune function. Consequently, GLR reflects both the body's sugar metabolism capacity and immune status, thereby helping predict NACT-related hematologic toxicity levels.

However, this study is associated with certain limitations. First, in the analysis of the correlation between body composition at the CT-L1 and L3 levels, the scarcity of patients for whom images describing the L3 level due to the absence of abdominal CT data among most BC patients necessitates further data validation. Second, owing to limitations associated with the sample size, we did not explore the GLR on hematologic toxicity in BC patients with diabetes or insulin resistance. Moreover, blood glucose levels fluctuate over time and in response to medications. The use of dexamethasone by some clinical practitioners before NACT may has a hyperglycemic effect, and the timing of measurements could introduce potential deviations. Therefore, the predictive capacity of GLR in hematologic toxicity requires further research for comprehensive validation. Finally, the defined cutoff value for sarcopenia in this study (32.91 cm^2^/m^2^) differs slightly from that described by Zhen Yu et al. (34.9 cm^2^/m^2^) for gastric cancer patients. Considering regional disparities, tumor discrepancies, and CT level variations, such marginal divergences in cutoff values are understandable. Nevertheless, further research is essential to explore the appropriate cutoff value for sarcopenia in Chinese BC patients.

In conclusion, this study underscores that in luminal-type BC patients, the CT-L1 level can effectively substitute for the L3 level for assessing body composition. Furthermore, a lower CT-L1 level skeletal muscle index is inversely correlated with severe hematologic toxicity during NACT. Additionally, tumor size and GLR serve as predictive factors for severe hematologic toxicity in luminal-type BC patients during NACT. Clinicians should identify and focus on such patients, closely monitoring relevant indicators during treatment and promptly performing interventions. Moreover, for controllable factors such as the skeletal muscle index and GLR levels, corresponding interventions (e.g. supplementation of high-quality protein, appropriate exercise) should be implemented before NACT to augment the skeletal muscle mass and diminish GLR levels, thereby reducing the risk of severe hematologic toxicity during NACT.

### Supplementary Information

Supplementary Table 1.

## Data Availability

Data is provided within the manuscript or supplementary information files.
