# Supplementary material for: Prediction of hematologic toxicity in luminal type breast cancer patients receiving neoadjuvant chemotherapy using CT L1 level skeletal muscle index
Source: Sci Rep. 2024 Apr 13;14:8604. doi: 10.1038/s41598-024-58433-9 (PMC11016056; doi:10.1038/s41598-024-58433-9)
Supplement: Supplementary file 1 — Supplementary Table 1. [file 41598_2024_58433_MOESM1_ESM.docx]

| Relationships between Body Composition, Related Indices, and Hematologic Toxicity | | | | |
| --- | --- | --- | --- | --- |
| Pathological Features | Groups | Univariate | Multivariate Logistic Regression Analysis | |
|  |  | p value | OR(95%CI) | p value |
| Age(years) | ≤49 | 0.141 |  |  |
|  | ＞49 |  |  |  |
| BMI(kg/m^2^) | ＜24 | 0.019 | 2.752(0.48-15.765) | 0.256 |
|  | ≥24 |  |  |  |
| Menopausal status | Menopausal | 0.487 |  |  |
|  | Non-menopausal |  |  |  |
| Initial Tumor Size(cm) | ＜3.335 | 0.819 |  |  |
|  | ≥3.335 |  |  |  |
| Occurrence of Axillary Lymph Node Metastasis | YES | 0.614 |  |  |
|  | NO |  |  |  |
| ER | - | 0.905 |  |  |
|  | + |  |  |  |
| PR | - | 0.722 |  |  |
|  | + |  |  |  |
| Ki67 | - | 0.774 |  |  |
|  | + |  |  |  |
| NLR | ＜1.86 | 0.221 |  |  |
|  | ≥1.86 |  |  |  |
| GLR | ＜2.88 | 0.009 | 6.649(1.56-28.342) | **0.01*** |
|  | ≥2.88 |  |  |  |
| SMA (cm²) | ＜87.32 | 0.003 |  |  |
|  | ≥87.32 |  |  |  |
| SMI (cm^2^/m^2^) | ＜32.9 | ＜0.001 | 0.258(0.074-0.899) | **0.033*** |
|  | ≥32.91 |  |  |  |
| IMFA (cm²) | ＜3.59 | 0.022 |  |  |
|  | ≥3.59 |  |  |  |
| IMFI (cm^2^/m^2^) | ＜1.35 | 0.022 | 0.526(0.111-2.504) | 0.42 |
|  | ≥1.35 |  |  |  |
| SFA (cm²) | ＜155.25 | 0.056 |  |  |
|  | ≥155.25 |  |  |  |
| SFI (cm^2^/m^2^) | ＜56.90 | 0.023 | 0.366(0.072-1.87) | 0.366 |
|  | ≥56.90 |  |  |  |
| VFA (cm²) | ＜112.55 | 0.046 |  |  |
|  | ≥112.55 |  |  |  |
| VFI (cm^2^/m^2^) | ＜34.78 | 0.015 | 0.27(0.055-1.323) | 0.106 |
|  | ≥34.78 |  |  |  |
